# Bioactive Cembranoids, Sarcocrassocolides P–R, from the Dongsha Atoll Soft Coral *Sarcophyton crassocaule*

**DOI:** 10.3390/md12020840

**Published:** 2014-01-28

**Authors:** Wan-Yu Lin, Bo-Wei Chen, Chiung-Yao Huang, Zhi-Hong Wen, Ping-Jyun Sung, Jui-Hsin Su, Chang-Feng Dai, Jyh-Horng Sheu

**Affiliations:** 1 Department of Marine Biotechnology and Resources, National Sun Yat-sen University, Kaohsiung 804, Taiwan; E-Mails: lemotylin@gmail.com (W.-Y.L.); a6152761@yahoo.com.tw (B.-W.C.); betty8575@yahoo.com.tw (C.-Y.H.); wzh@mail.nsysu.edu.tw (Z.-H.W.); 2 National Museum of Marine Biology and Aquarium, Pingtung 944, Taiwan; E-Mails: pjsung@nmmba.gov.tw (P.-J.S.); x2219@nmmba.gov.tw (J.-H.S.); 3 Graduate Institute of Marine Biotechnology and Department of Life Science and Institute of Biotechnology, National Dong Hwa University, Pingtung 944, Taiwan; 4 Institute of Oceanography, National Taiwan University, Taipei 112, Taiwan; E-Mail: corallab@ntu.edu.tw; 5 Graduate Institute of Natural Products, Kaohsiung Medical University, Kaohsiung 807, Taiwan; 6 Department of Medical Research, China Medical University Hospital, China Medical University, Taichung 404, Taiwan; 7 Asia Pacific Ocean Research Center, National Sun Yat-sen University, Kaohsiung 804, Taiwan; 8 Frontier Center for Ocean Science and Technology, National Sun Yat-sen University, Kaohsiung 804, Taiwan

**Keywords:** soft coral, *Sarcophyton crassocaule*, cytotoxic activity, anti-inflammatory activity

## Abstract

New cembranoids, sarcocrassocolides P–R (**1**–**3**) and four known compounds (**4**–**7**) were isolated from the soft coral *Sarcophyton crassocaule*. The structures of the metabolites were determined by extensive spectroscopic analysis. Compounds **3**–**5** and **7** were shown to exhibit cytotoxicity toward a limited panel of cancer cell lines and all compounds **1**–**7** displayed potent *in vitro* anti-inflammatory activity in lipopolysaccharide (LPS)-stimulated RAW264.7 macrophage cells by inhibiting the expression of inducible nitric oxide synthase (iNOS) protein. Compound **7** also showed significant activity in reducing the accumulation of cyclooxygenase-2 (COX-2) protein in the same macrophage cells.

## 1. Introduction

**Chart 1:**
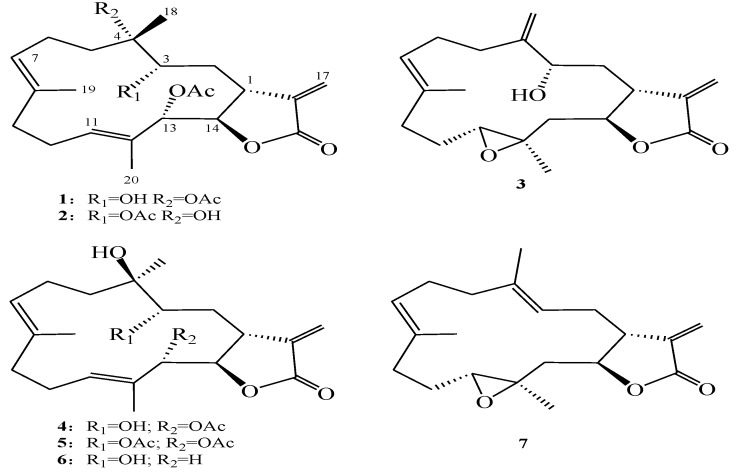
Structures of new metabolites **1**–**3**, and known compounds **4**–**7**.

Marine terpenoids are of considerable interest due to their unique structures and wide range of biological activities [1]. The macrocyclic cembrane-derived compounds are known to be the major diterpenoidal metabolites in soft corals [2,3,4,5,6,7,8,9]. In previous studies of the secondary metabolites from soft corals of Taiwan waters, a series of bioactive cembranoids was discovered from soft corals belonging to the genera *Sinularia* [10,11,12,13,14,15,16,17], *Lobophytum* [18,19,20,21], *Sarcophyton* [22,23,24,25,26,27,28] and *Pachyclavularia* [29]. Some of these metabolites have been shown to exhibit cytotoxic activity against the growth of various cancer cell lines [11,12,13,15,17,19,20,21,22,23,24,25,26,27,28], and/or anti-inflammatory activity [10,11,14,15,16,17,18,19]. Our previous studies on the chemical constituents of a Dongsha Atoll soft coral *S. crassocaule* have yielded 15 new cembranoids, sarcocrassocolides A–O, of which several compounds were shown to exhibit significant cytotoxic and anti-inflammatory activities [30,31,32]. Our continuing chemical study on the same collection of this organism again led to the isolation of three new cembranoids, sarcocrassocolides P–R (**1**–**3**) (Chart 1 and Supplementary Figures S1–S9) along with four known compounds, crassocolides A, B, D, and E (**4**–**7**) [23] (Chart 1). The structures of **1**–**3** were established by extensive spectroscopic analysis, including careful examination of 2D NMR (^1^H–^1^H COSY, HSQC, HMBC and NOESY) correlations. The cytotoxicity of compounds **1**–**7** against human colon adenocarcinoma (DLD-1), human T-cell acute lymphoblastic leukemia (CCRF-CEM), and human promyelocytic leukemia (HL-60) cell lines was studied, and the ability of **1**–**7** to inhibit the up-regulation of pro-inflammatory iNOS (inducible nitric oxide synthase) and COX-2 (cyclooxygenase-2) proteins in LPS (lipopolysaccharide)-stimulated RAW264.7 macrophage cells was also examined. Compounds **1**–**7** were shown to exhibit cytotoxicity towards the above cancer cells, with **5** being the most cytotoxic. 

## 2. Results and Discussion

**Figure 1:**
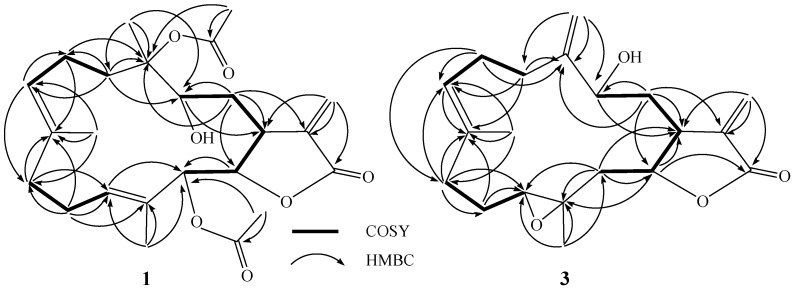
COSY and HMBC correlations for **1** and **3**.

**Figure 2:**
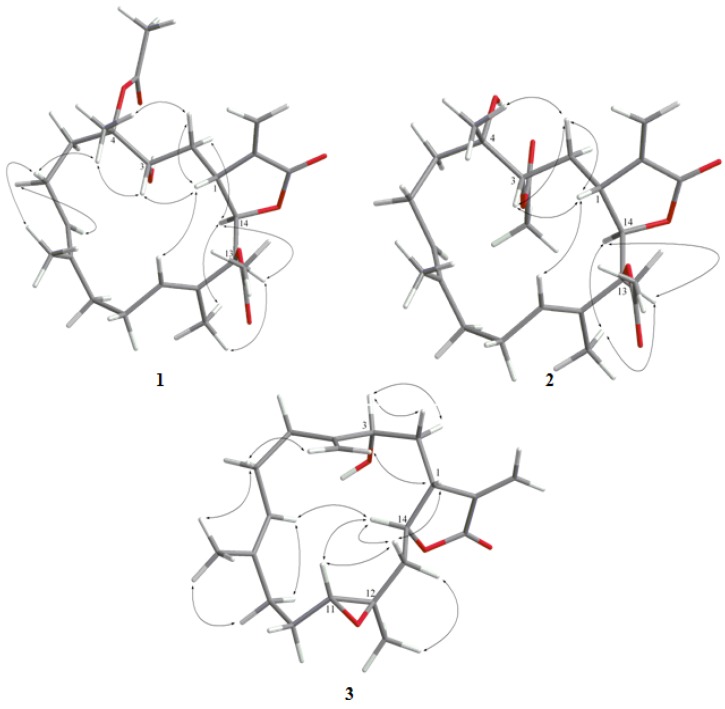
Key NOESY correlations for **1**–**3**.

The HRESIMS spectrum of sarcrocrassocolide P (**1**) established the molecular formula C_2__4_H_3__4_O_7_, appropriate for eight degrees of unsaturation, and the IR spectrum revealed the presence of a hydroxyl (3445 cm^−1^) and carbonyl (1767 cm^−1^) group. The ^13^C NMR and DEPT (Distortionless Enhancement by Polarization Transfer) (Table 1) spectroscopic data showed signals of five methyls (including two acetate methyls), five sp^3^ methylenes, one sp^2^ methylene, four sp^3^ methines (including three oxymethines), two sp^2^ methines, one sp^3^ and six sp^2^ quaternary carbons (including two ester carbonyls). The NMR signals (Table 1) at δ_C_ 170.1 (C), 140.5 (C), 120.9 (CH_2_), 79.1 (CH), and 38.5 (CH), and δ_H_ 6.24, 5.65 (each, 1H, d, *J* = 2.0 Hz), 5.28 (1H, brs), and 3.11 (1H, d, *J* = 9.5 Hz) showed the presence of an α-methylene-γ-lactonic group by comparing with the NMR data of known cembranoids with the same five-membered lactone ring [30,31,32]. Two trisubstituted double bonds were also identified from NMR signals appearing at δ_C_ 135.8 (C), 125.7 (CH) and δ_H_ 5.08 (1H, t, *J* = 7.0 Hz), and at δ_C_ 130.3 (C), 127.3 (CH) and δ_H_ 5.32 (1H, dd, *J* = 10.0, 3.5 Hz), respectively. In the COSY spectrum, it was possible to identify three partial structures, which were assembled with the assistance of an HMBC experiment. Key HMBC correlations of H_3_-18 to C-3, C-4 and C-5; H_3_-19 to C-7, C-8 and C-9; H_3_-20 to C-11, C-12 and C-13 and H_2_-17 to C-1, C-15 and C-16 permitted the establishment of the carbon skeleton (Figure 1). Furthermore, the acetoxy group positioned at C-13 was confirmed from the HMBC correlations of the methyl protons of an acetate (δ_H_ 1.99) to the ester carbonyl carbon at δ_C_ 169.3 and the oxymethine signal at 77.5 (C-13, CH). The downfield chemical shift for H_3_-18 (δ 1.44 s) and the ^13^C NMR signals at δ_C_ 89.9 (C) showed the presence of an acetate group at C-4. The geometries of trisubstituted double bonds at C-7/C-8 and C-11/C-12 are both *E*, as the chemical shifts for C-19 and C-20 were upfield shifted to 16.0 and 14.5 ppm. On the basis of the above analysis, the planar structure of **1** was established. The relative structure of **1 **was elucidated by the NOE correlations, as shown in Figure 2. The NOE interaction of H-1 (δ 3.11) with H-3 (δ 3.73) and H-11 (δ 5.32) revealed the β*-*orientation of H-1 and H-3 [23,30,31,32]. H-3 showed NOE correlation with H_3_-18 (δ 1.32, s), thus H_3_-18 should also be positioned on the β*-*face. The *E* geometry of the trisubstituted double bonds at C-7/C-8 and C-11/C-12 were confirmed from the NOE correlations of H_3_-19 (δ 1.67) with one proton of H_2_-6 (δ 2.26), and H_3_-20 with H-10. H-14 (δ 5.28) exhibited NOE correlations with both H-13 (δ 5.40) and H_3_-20, but not with H-1, indicating the *α*-orientation of both H-13 and H-14. These results, together with other detailed NOE correlations of **1** (Figure 2), unambiguously established the structure of sarcocrassocolide P, as shown in formula **1** (Chart 1). Therefore, the relative stereochemistry of compound **1** was determined.

**Table 1:** NMR spectroscopicdata for Sarcrocrassocolides M–O(**1**–**3**).

Sarcrocrassocolide P (1)			Sarcrocrassocolide Q (2)		Sarcrocrassocolide R (3)	
position	δ_C_, mult. ^a^	δ_H_ (*J* in Hz) ^b^	δ_C_, mult. ^a^	δ_H_ (*J* in Hz) ^b^	δ_C_, mult. ^c^	δ_H_ (*J* in Hz) ^d^
1	38.5, CH	3.11, brd (9.5) ^c^	37.7, CH	3.06, brs	40.5, CH	3.02, d (11.0)
2	37.3, CH_2_	1.80, m	35.7, CH_2_	2.05, t (5.0)	39.3, CH_2_	2.14, m
		1.32, ddd (14.5, 10.5, 9.5)		1.80, m		1.82, ddd (19.0, 5.5, 1.5)
3	73.1, CH	3.73, t (10.0)	75.8, CH	5.04, dd (6.5, 5.0)	71.6, CH	4.25, d (5.0)
4	89.9, C		74.7, C		150.5, C	
5	36.4, CH_2_	1.94, t (11.5)	37.9, CH_2_	1.68, m	31.2, CH_2_	2.16, m
		1.81, m				2.12, m
6	23.1, CH_2_	2.26, m	23.1, CH_2_	2.18, m	23.4, CH_2_	2.59, m
		2.15, m				2.21, m
7	125.7, CH	5.08, t (7.0)	123.3, CH	5.13, t (7.0)	126.3, CH	5.07, d (10.5)
8	135.8, C		136.5, C		133.9, C	
9	39.4, CH_2_	2.28, m	37.6, CH_2_	2.21, m	36.7, CH_2_	2.29, d (13.0)
		2.09, m				2.09, m
10	24.7, CH_2_	2.44, qd (10.0, 2.5)	24.6, CH_2_	2.34, m	24.4, CH_2_	2.17, m
		2.11, m		2.24, m		1.31, m
11	127.3, CH	5.32, dd (10.0, 3.5)	129.3, CH	5.32, brt (7.0)	61.7, CH	2.56, dd (11.0, 4.0)
12	130.3, C		129.2, C		59.7, C	
13	77.5, CH	5.40, s	76.5, CH	5.37, s	46.5, CH_2_	2.00, dd (14.0, 11.5)
						1.24, d (14.0)
14	79.1, CH	5.28, brs	82.7, CH	4.43, dd (5.0, 2.0)	81.1, CH	4.32, d (11.5)
15	140.5, C		140.5, C		139.5, C	
16	170.1, C		169.8, C		170.1, C	
17	120.9, CH_2_	6.24, d (2.0)	122.2, CH_2_	6.23, d (2.5)	123.2, CH_2_	6.29, d (1.5)
		5.65, d (2.0)		5.78, d (2.5)		5.69, d (1.5)
18	19.7, CH_3_	1.44, s	24.4, CH_3_	1.44, s	107.3, CH_2_	5.17, s
						4.78, s
19	16.0, CH_3_	1.67, s	16.8, CH_3_	1.65, s	14.9, CH_3_	1.76, s
20	14.5, CH_3_	1.72, s	14.6, CH_3_	1.72, s	17.5, CH_3_	1.38, s
4-OAc	22.1, CH_3_	2.04, s				
	172.1, C					
3-OAc			21.2, CH_3_	2.08, s		
			170.4, C			
13-OAc	20.8, CH_3_	1.99, s	20.8, CH_3_	2.03, s		
	169.3, C		169.5, C			

^a^ Spectra recorded at 125 MHz in CDCl_3_; ^b^ Spectra recorded at 500 MHz in CDCl_3_; ^c^ Spectra recorded at 100 MHz in CDCl_3_; ^d^ Spectra recorded at 400 MHz in CDCl_3_.

Sarcocrassocolide Q (**2**), with a molecular formula of C_24_H_34_O_7_, was obtained as a colorless oil. Comparison of its ^1^H and ^13^C NMR data with those of **1** suggested that **2 **has the same molecular formula, and showed that a hydroxy group at C-3 and the acetoxy group at C-4 in **1 **were replaced by an acetoxy and hydroxy group in **2**, respectively, as confirmed by the downfield shifted δ value of C-3 (δ_C_ 73.1) of **1**, relative to that of **2** (δ_C_ 75.8), and the HMBC correlation from H-3 (δ 5.04) to the carbonyl carbon resonating at δ 170.4. The *E* geometry of the trisubstituted double bonds at C-7/C-8 and C-11/C-12 were assigned from the upper field chemical shift of C-19 (δ 16.8) and C-20 (δ 14.6). Further analysis of the NOE interactions revealed that **2** possessed the same relative configurations at C-1, C-3, C-4, C-13, and C-14 as those of **1** (Figure 2).

Compound **3** was shown by HRESIMS to possess the molecular formula C_20_H_28_O_4_ (*m/z* 355.1888 [M + Na]^+^). The IR spectrum of **3** also revealed the presence of hydroxy (3420 cm^−1^) and carbonyl (1752 cm^−1^) groups. Comparison of the ^1^H and ^13^C NMR data (Table 1) of compounds **3** and that of crassocolide E showed that the structure of **3** has some similarity to that of crassocolide E [23]. It was found that a C-3/C-4 double bond in crassocolide E was replaced by a 1,1-disubstituted carbon–carbon double bond at C-4/C-18 and a hydroxy group at C-3 in **3**, as confirmed by HMBC correlations observed from H_2_-18 to C-3 (δ_C_ 71.6), C-4 (δ_C_ 150.5), and C-5 (δ_C_ 31.2). The planar structure of **3** was elucidated by analyzing the COSY and HMBC correlations (Figure 1). The relative stereochemistry of **3** was confirmed from the key NOESY correlations (Figure 2). Assuming the β*-*orientation of H-1, correlations of H-1 with both of one proton of H_2_-18 (δ 5.17) and one proton of H_2_-13, which was assigned as H-13β (δ 1.24), but not with H-3; H-13β with H-11 (δ 2.56); H-11 with H-14 (δ 4.32); H_3_-20 with H-13*α* (δ 2.00); and one proton of H-9 (δ 2.09) with H-7, which did not show NOE correlation with H_3_-19, revealed the β*-*orientations of H-1 and H-11, the *α*-orientation of H-14, the *E* geometry of the trisubstituted double bond, and the *trans* stereochemistry of 11,12-epoxide. These results, together with other detailed NOE correlations of **3**, established the structure of sarcocrassocolide R, as shown in formula **3 **(Chart 1). 

Known compounds **4**–**7** (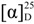 +7.0, +31.6, +21.9 and +108.9, respectively), were found to have identical spectroscopic data and close specific optical rotations with those of previously discovered compounds, crassocolide A (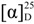 +6.5), B (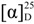 +26.5), D (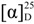 +16.8) and E (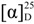 +99.6), respectively [23]. Thus, the structures of compounds **4**–**7 **were confirmed.

**Figure 3:**
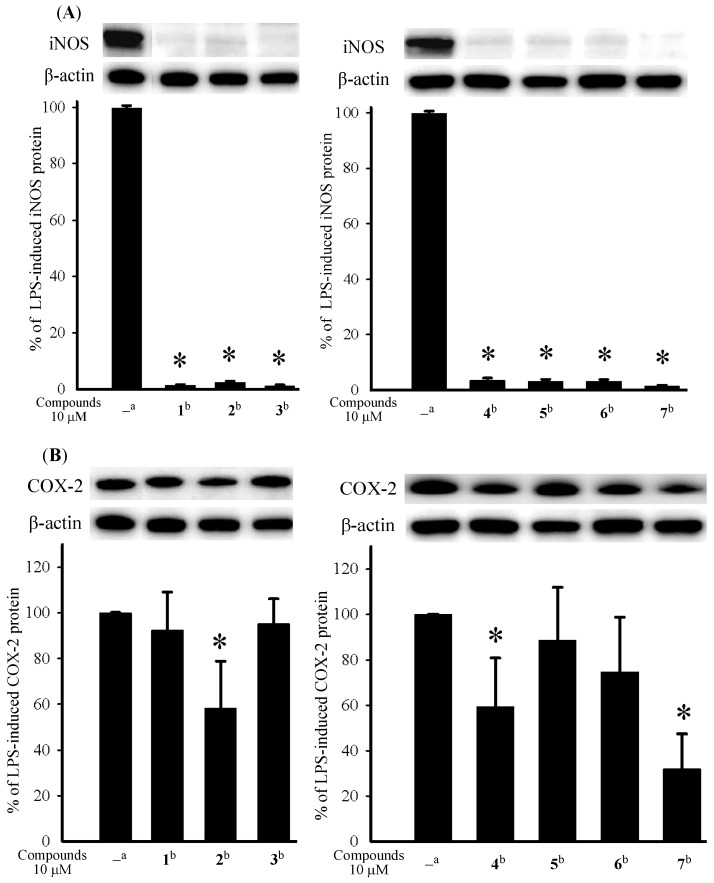
Effect of compounds **1**–**7** on the expression of inducible nitric oxide synthase (iNOS) and cyclooxygenase-2 (COX-2) proteins in RAW264.7 macrophage cells by immunoblot analysis. (**A**) Immunoblots of iNOS and β-actin; (**B**) Immunoblots of COX-2 and β-actin. The values are mean ± SEM. (*n* = 6). Relative intensity of the lipopolysaccharide (LPS) alone stimulated group was taken as 100%; * Significantly different from LPS alone stimulated group (* *p* < 0.05); ^a^ stimulated with LPS; ^b^ stimulated with LPS in the presence of **1**–**7** (10 µM).

The cytotoxicity of compounds **1**–**7** against the proliferation of a limited panel of cancer cell lines, including DLD-1, CCRF-CEM, and HL-60 carcinoma cell lines was evaluated. The results (Table 2) showed that all compounds **3**–**5**, **7** were found to exhibit significant cytotoxicity against all or part of the above carcinoma cell lines. Compound **5** was found to be the most cytotoxic. The inhibition of LPS-induced up-regulation of pro-inflammatory proteins iNOS and COX-2 in RAW264.7 macrophage cells was measured by immunoblot analysis (Figure 3). At a concentration of 10 µM of each compound, **1**–**7** were found to potently reduce the levels of iNOS protein to 1.3% ± 0.3%, 2.4% ± 0.4%, 1.2% ± 0.3%, 3.5% ± 0.9%, 3.2% ± 0.7%, 3.2% ± 0.6%, and 1.4% ± 0.4% respectively, relative to the control cells stimulated with LPS only. At the same concentration metabolites **1**, **3**, **5**, and **6** did not show activity in inhibiting the expression of the pro-inflammatory COX-2 protein with LPS treatment, but compounds **2**, **4**, and **7** could reduce the expression of COX-2 to 58.3% ± 20.5%, 59.4% ± 21.4%, and 32.0% ± 15.3%. Thus, compounds **1**–**7** might be useful anti-inflammatory agents, while **7** could be regarded as a promising COX-2 inhibitor. Compounds **3**–**5** and **7**, in particular **5**, are worthy of further anticancer studies.

**Table 2:** Cytotoxicity (ED_50_ µM) of compounds **1**–**3**.

Compound	DLD-1 ^a^	CCRF-CEM ^b^	HL-60 ^c^
**1**	21.8	48.8	24.9
**2**	35.8	73.1	18.6
**3**	10.0	28.1	8.7
**4**	5.7	6.3	(–) ^d^
**5**	3.8	8.7	7.3
**6**	27.7	41.9	34.6
**7**	7.9	11.1	8.4
Doxorubicin	0.77	1.16	0.046

^a^ DLD-1: human colon adenocarcinoma; ^b^ CCRF-CEM: human T-cell acute lymphoblastic leukaemia; ^c^ HL-60: human promyelocytic leukemia; ^d^ (–): ED_50_ > 50 µM.

## 3. Experimental Section

### 3.1. General Experimental Procedures

Optical rotations were measured on a JASCO P-1020 polarimeter. Ultraviolet spectra were recorded on a JASCO V-650 spectrophotometer. IR spectra were recorded on a JASCO FT/IR-4100 infrared spectrophotometer. NMR spectra were recorded on a Varian 400MR FT-NMR (or Varian Unity INOVA500 FT-NMR) instrument at 400 MHz (or 500 MHz) for ^1^H and 100 MHz (or 125 MHz) for ^13^C in CDCl_3_. LRMS and HRMS were obtained by ESI on a Bruker APEX II mass spectrometer. Silica gel (Merck, 230–400 mesh) was used for column chromatography. Precoated silica gel plates (Merck, Kieselgel 60 F-254, 0.2 mm) were used for analytical TLC. High-performance liquid chromatography was performed on a Hitachi L-7100 HPLC apparatus with a Merck Hibar Si-60 column (250 × 21 mm, 7 µm) and on a Hitachi L-2455 HPLC apparatus with a Supelco C18 column (250 × 21.2 mm, 5 µm).

### 3.2. Animal Material

*S. crassocaule* (specimen No. 20070402) was collected by hand, using scuba off the coast of Dongsha, Taiwan, in April 2007, at a depth of 5–10 m, and stored in a freezer until extraction. A voucher sample was deposited at the Department of Marine Biotechnology and Resources, National Sun Yat-sen University.

### 3.3. Extraction and Separation

The frozen bodies of S. crassocaule (0.5 kg, wet wt) were minced and exhaustively extracted with EtOAc (1 L × 5). The EtOAc extract (7.3 g) was chromatographed over silica gel by column chromatography and eluted with EtOAc in n-hexane (0%–100%, stepwise) then with acetone in EtOAc (50%–100%, stepwise) to yield 28 fractions. Fraction 10, eluting with *n*-hexane–EtOAc (6:1), was further purified over silica gel using *n*-hexane–acetone (7:1) to afford six subfractions (A1–A5). Subfraction A3 was separated by normal-phase HPLC using CH_2_Cl_2_–Acetone (40:1) to afford **7 **(79.8 mg). Fraction 15, eluting with *n*-hexane–EtOAc (2:1), was further purified over silica gel using *n*-hexane–acetone (3:1) to afford six subfractions (B1–B5). Subfraction B4 was separated by reverse-phase HPLC using MeOH–H_2_O (2.3:1) to afford **1 **(5.8 mg). Fraction 18, eluting with *n*-hexane–EtOAc (1:1), was further purified over silica gel using *n*-hexane–acetone (3:1) to afford eight subfractions (C1–C6). Subfraction C6 was separated by reversed-phase HPLC using MeOH–H_2_O (1.5:1 and 1.2:1) to afford **2** (1.5 mg), **3** (1.6 mg), **4** (3.5 mg), **5** (4.3 mg), and **6** (13.8 mg). 

Sarcocrassocolide P (**1**): colorless oil; 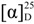 −76 (*c* 0.4, CHCl_3_); IR (neat) ν_max_ 3445, 2924, 2851, 1767, 1733, 1652, 1435, 1371, and 1229 cm^−^^1^; UV (MeOH) λ_max_ 205 (log ε = 3.5); ^13^C and ^1^H NMR data, see Table 1; ESIMS *m/z* 457 [M + Na]^+^; HRESIMS *m/z* 457.2199 [M + Na]^+^ (calcd. for C_24_H_34_O_7_Na, 457.2202).

Sarcocrassocolide Q (**2**): colorless oil; 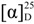 −84 (*c* 0.1, CHCl_3_); IR (neat) ν_max_ 3445, 2917, 2849, 1750, 1733, 1653, 1434, 1372, and 1236 cm^−^^1^; UV (MeOH) λ_max_ 214 (log ε = 3.8); ^13^C and ^1^H NMR data, see Table 1; ESIMS *m/z* 457 [M + Na]^+^; HRESIMS *m/z* 457.2201 [M + Na]^+^ (calcd. for C_24_H_34_O_7_Na, 457.2202).

Sarcocrassocolide R (**3**): colorless oil; 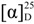 −178 (*c* 0.1, CHCl_3_); IR (neat) ν_max_ 3420, 2931, 1751, 1654, 1450, 1375, and 1270 cm^−^^1^; UV (MeOH) λ_max_ 213 (log ε = 3.7); ^13^C and ^1^H NMR data, see Table 1; ESIMS *m/z* 355 [M + Na]^+^; HRESIMS *m/z* 355.1888 [M + Na]^+^ (calcd. for C_20_H_28_O_4_Na, 355.1885).

### 3.4. Cytotoxicity Testing

Cell lines were purchased from the American Type Culture Collection (ATCC). Cytotoxicity assays of the tested compounds **1**–**7** were performed using the MTT [3-(4,5-dimethylthiazol-2-yl)-2,5-diphenyltetrazolium bromide] colorimetric method [33]. To measure the cytotoxicity activities of tested compounds, three concentrations in DMSO with three replications were performed on each cell line. Doxorubicin and DMSO were used as positive and negative controls, respectively in this assay.

### 3.5. *In Vitro* Anti-Inflammatory Assay

Macrophage (RAW264.7) cells were purchased from ATCC. *In vitro* anti-inflammatory activities of compounds **1**–**7** were measured by examining the inhibition of lipopolysaccharide (LPS) induced upregulation of iNOS (inducible nitric oxide synthetase) and COX-2 (cyclooxygenase-2) proteins in macrophages cells using western blotting analysis [34]. For statistical analysis, all of the data were analyzed by a one-way analysis of variance (ANOVA), followed by the Student-Newman-Keuls *post hoc* test for multiple comparisons. A significant difference was defined as a *p* value of <0.05.

## 4. Conclusions

Our investigation demonstrated that the soft coral, *S*. *crassocaule*, is a good source of bioactive substances. Compounds **1**–**7**, in particular **7**, are potentially anti-inflammatory and may become lead compounds in future anti-inflammation drug development. Compounds **3**–**5**, and **7**, in particular **5**, are worthy of further anticancer studies. These results suggest that continuing investigation of novel secondary metabolites together with the potentially useful bioactivities from this marine organism are worthwhile for future drug development.

## Supplementary Files

Supplementary File 1 Supplementary Information (PDF, 434 KB)
