# Supplementary material for: Bioactive Cembranoids, Sarcocrassocolides P–R, from the Dongsha Atoll Soft Coral Sarcophyton crassocaule
Source: Mar Drugs. 2014 Jan 28;12(2):840–50. doi: 10.3390/md12020840 (PMC3944518; doi:10.3390/md12020840)
Supplement: Supplementary File 1 — Supplementary Information (PDF, 434 KB) [file marinedrugs-12-00840-s001.pdf]

# Supplementary Information

**Figure S1.** $^{1}$H NMR spectrum of **1** in CDCl3 at 500 MHz.

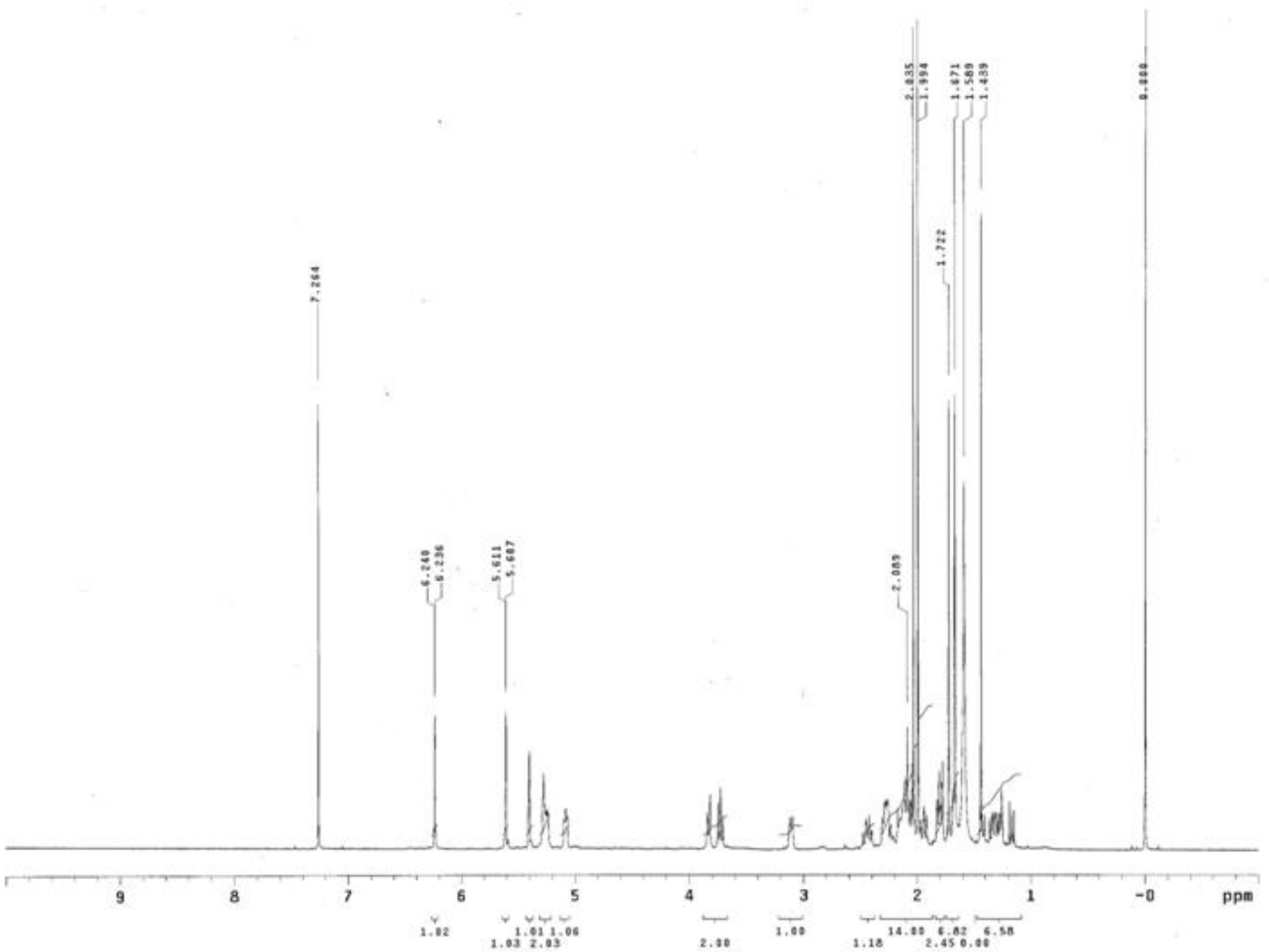

**Figure S2.** $^{1}$H NMR spectrum (1.3–6.3 ppm) of **1** in CDCl3 at 500 MHz.

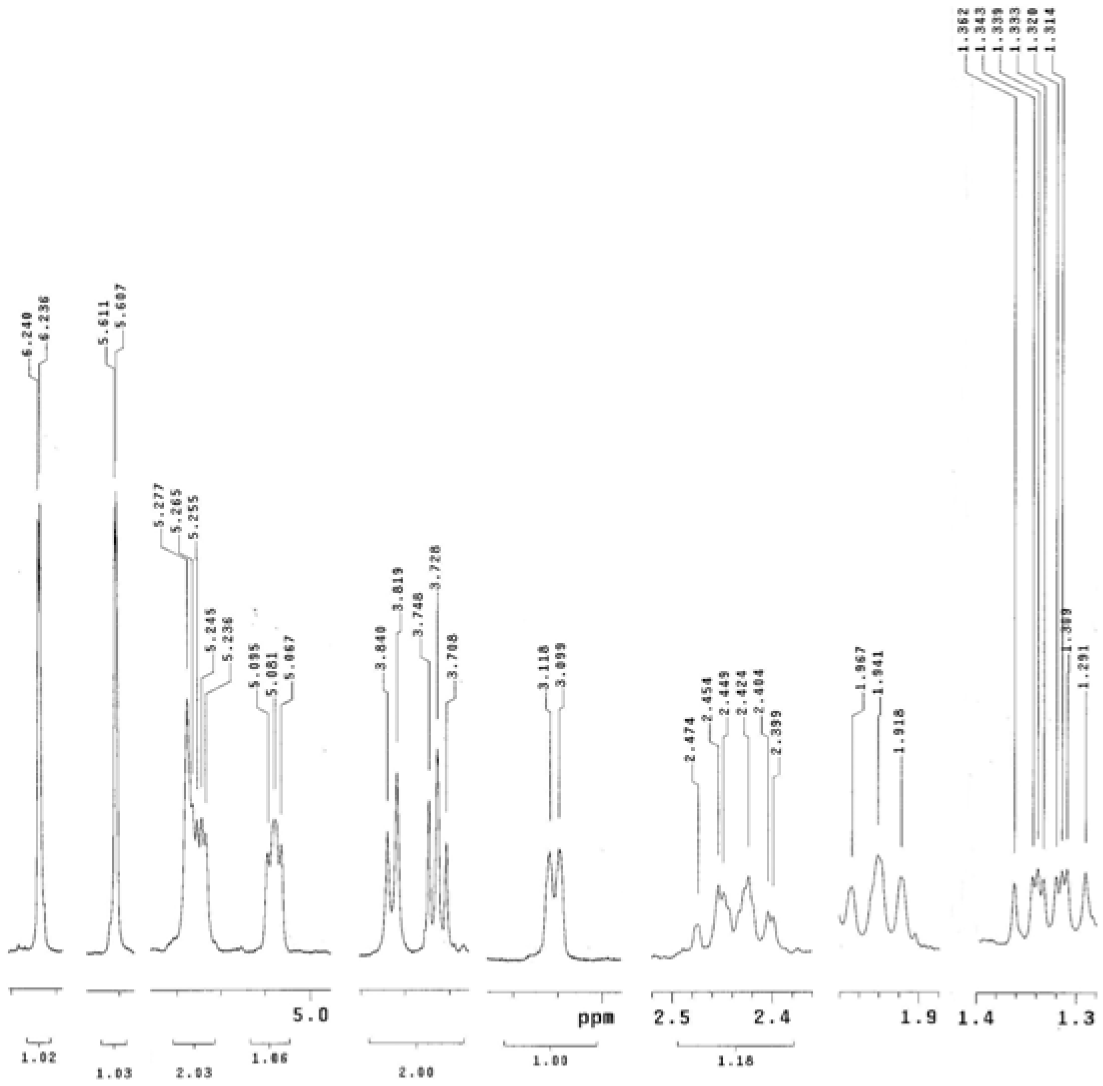

**Figure S3.** $^{13}$C NMR spectrum of **1** in CDCl3 at 125 MHz.

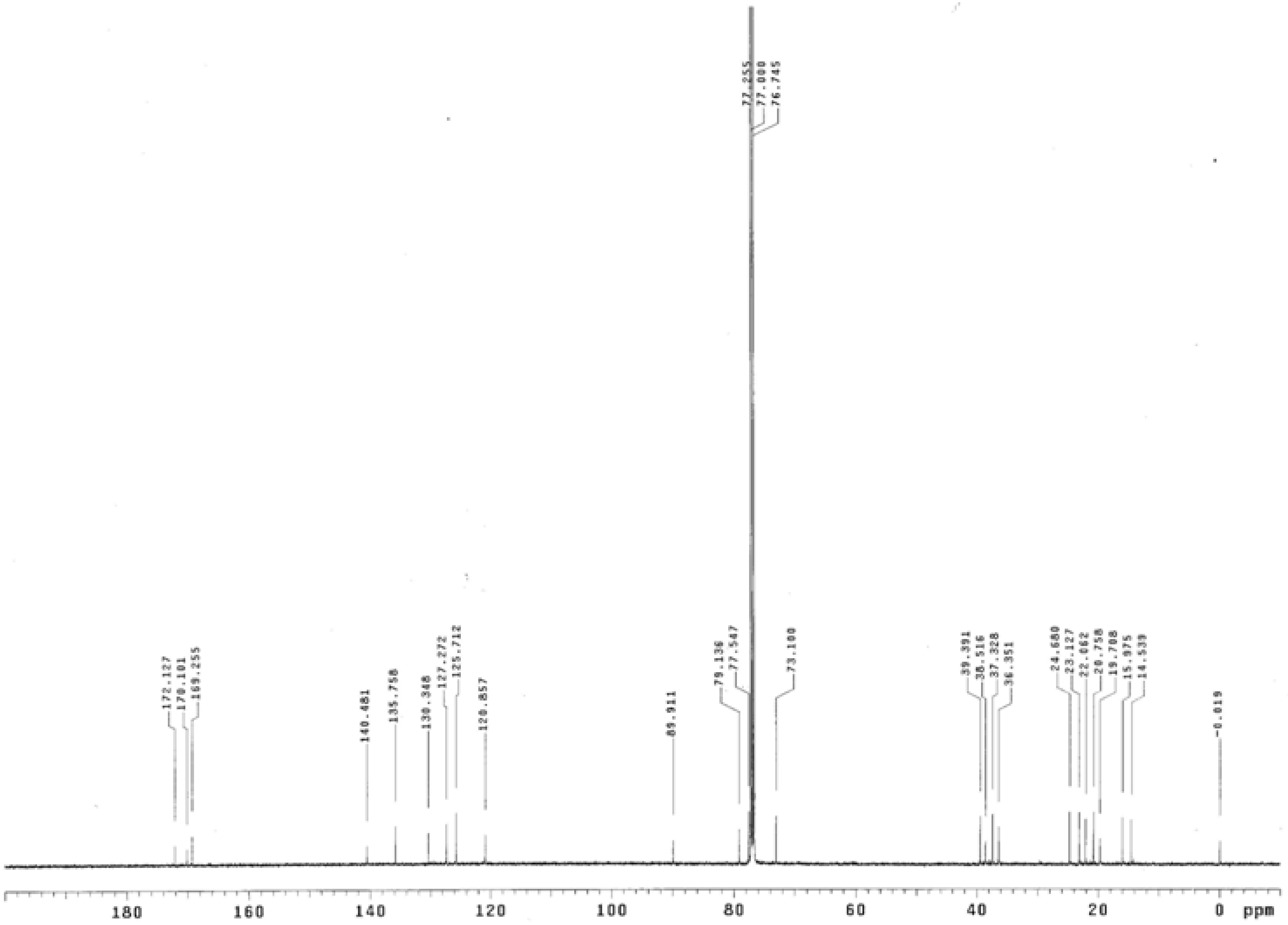

**Figure S4.** $^{1}$H NMR spectrum of **2** in CDCl3 at 500 MHz.

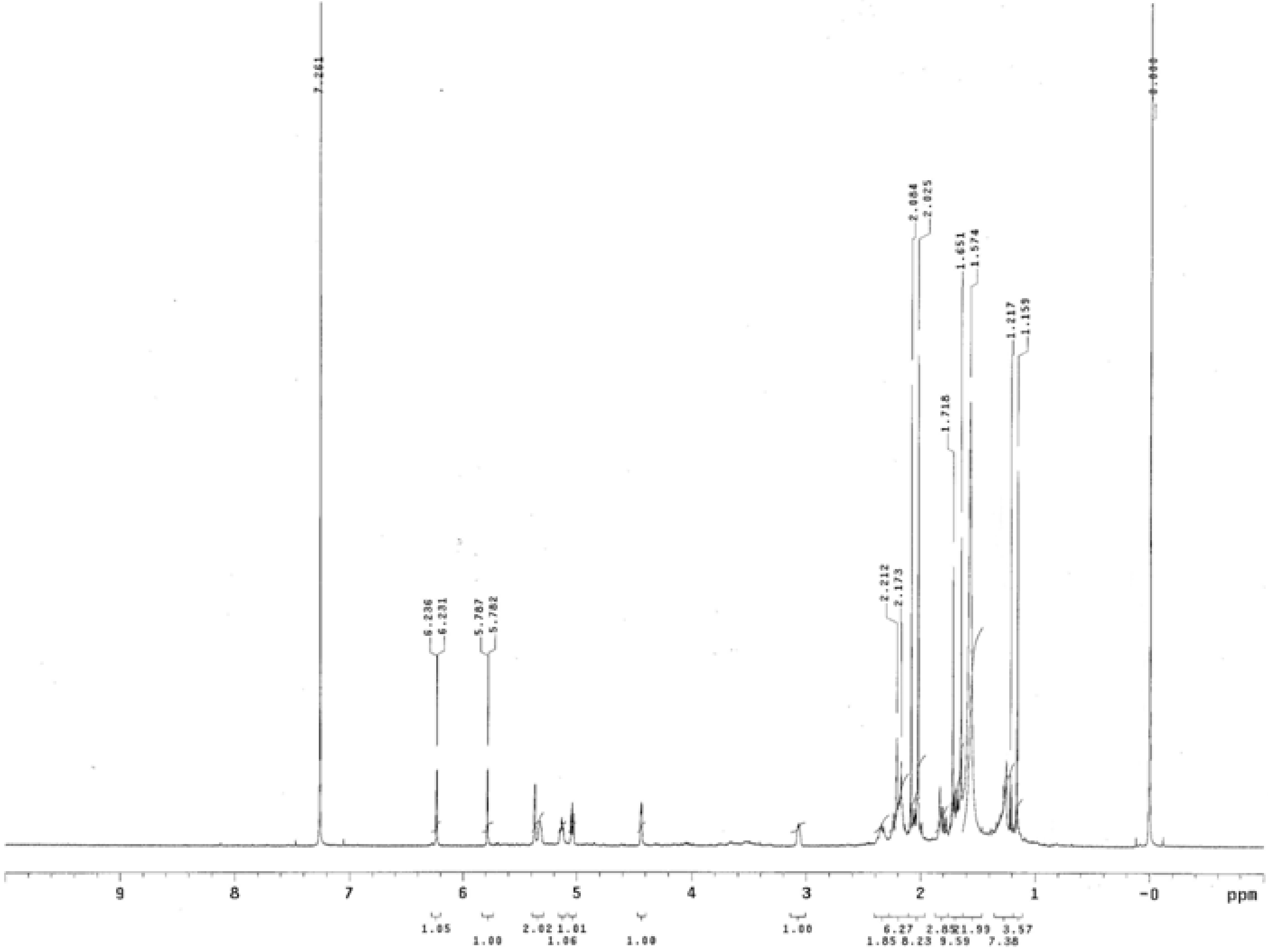

**Figure S5.** $^{1}$H NMR spectrum (1.8–6.3 ppm) of **2** in CDCl3 at 500 MHz.

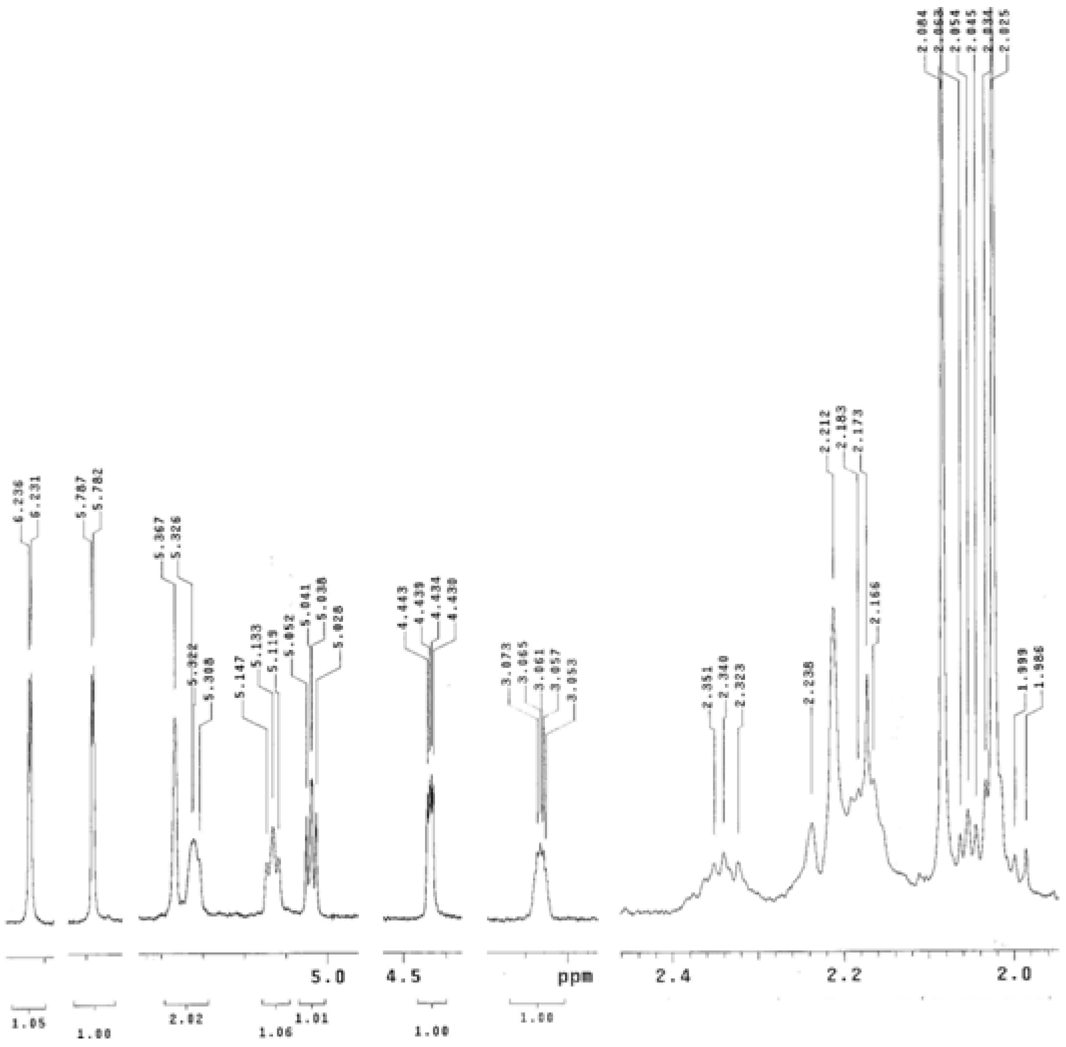

**Figure S6.** $^{13}$C NMR spectrum of **2** in CDCl3 at 125 MHz.

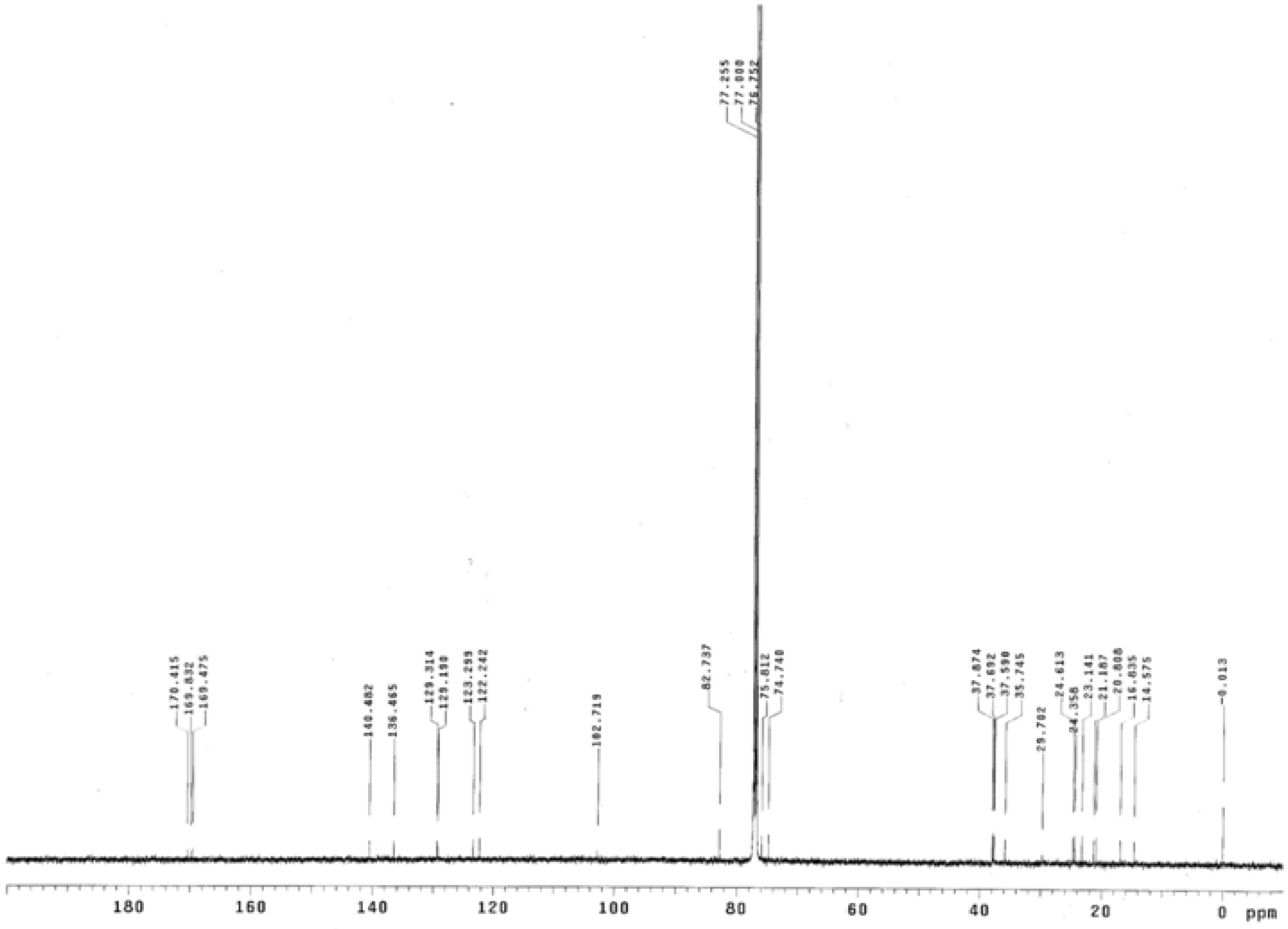

**Figure S7.** $^{1}$H NMR spectrum of **3** in CDCl3 at 500 MHz.

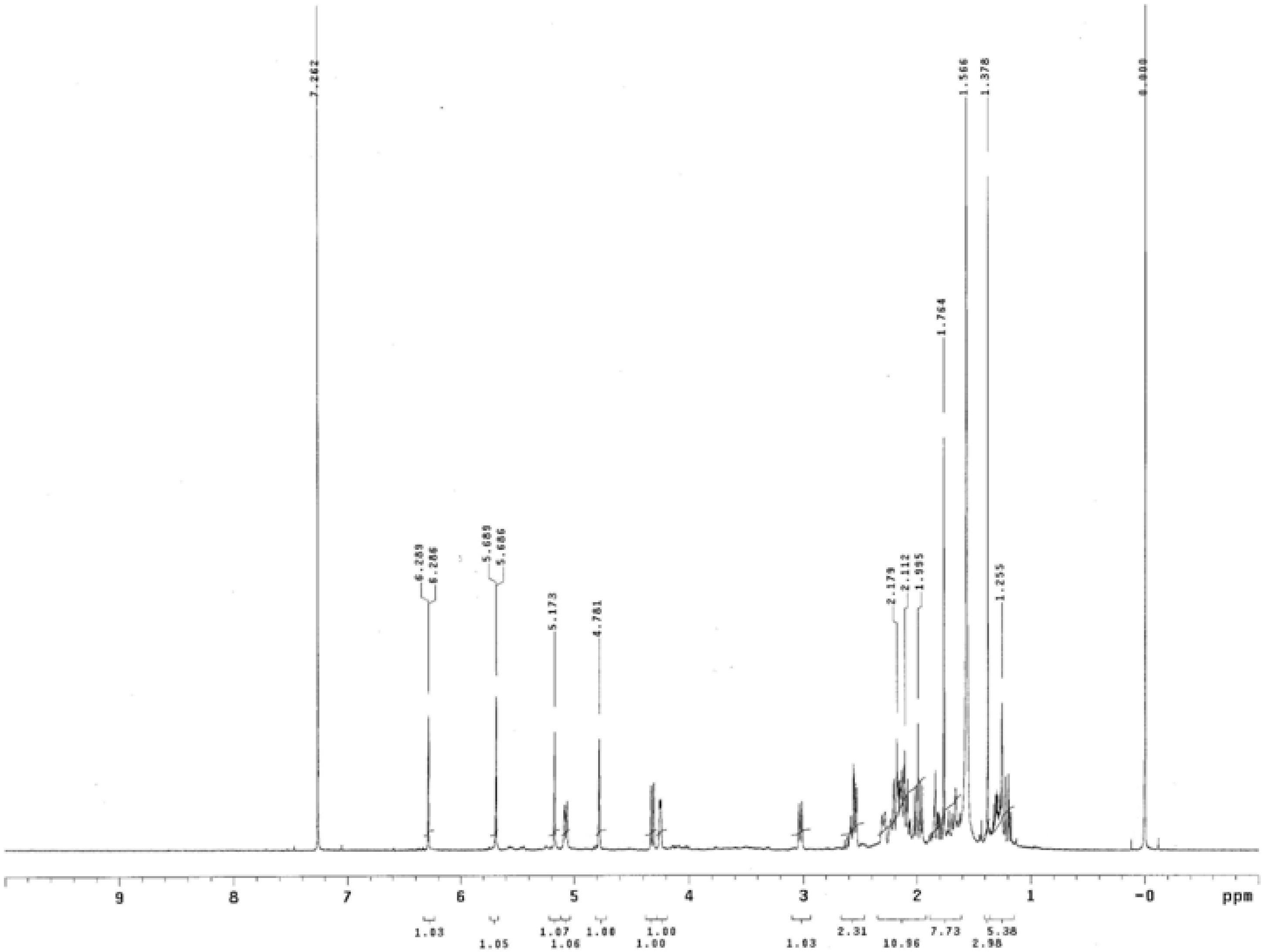

**Figure S8.** $^{1}$H NMR spectrum (1.1–6.3 ppm) of **3** in CDCl3 at 500 MHz.

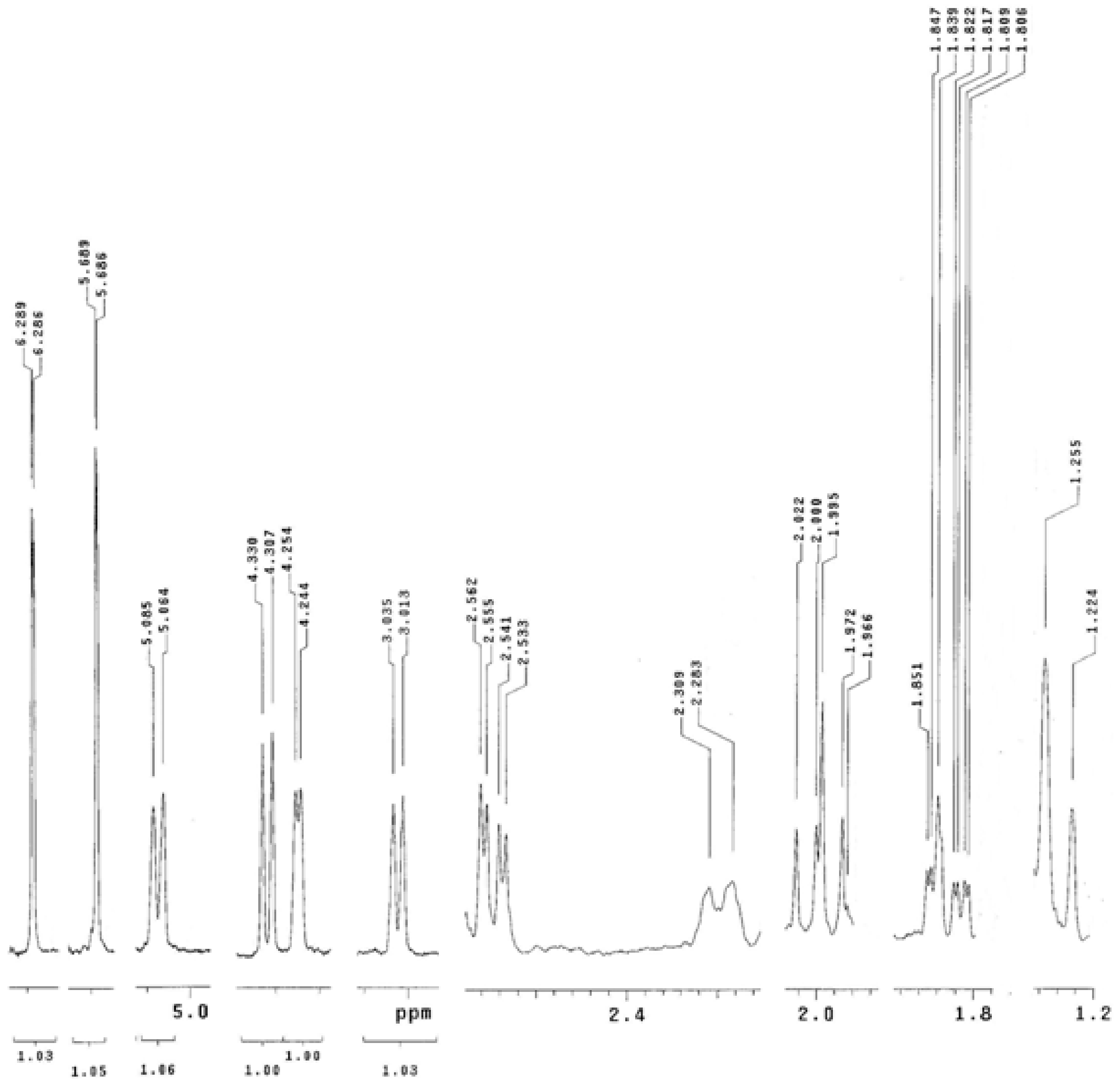

**Figure S9.** $^{13}$C NMR spectrum of **3** in CDCl3 at 125 MHz.

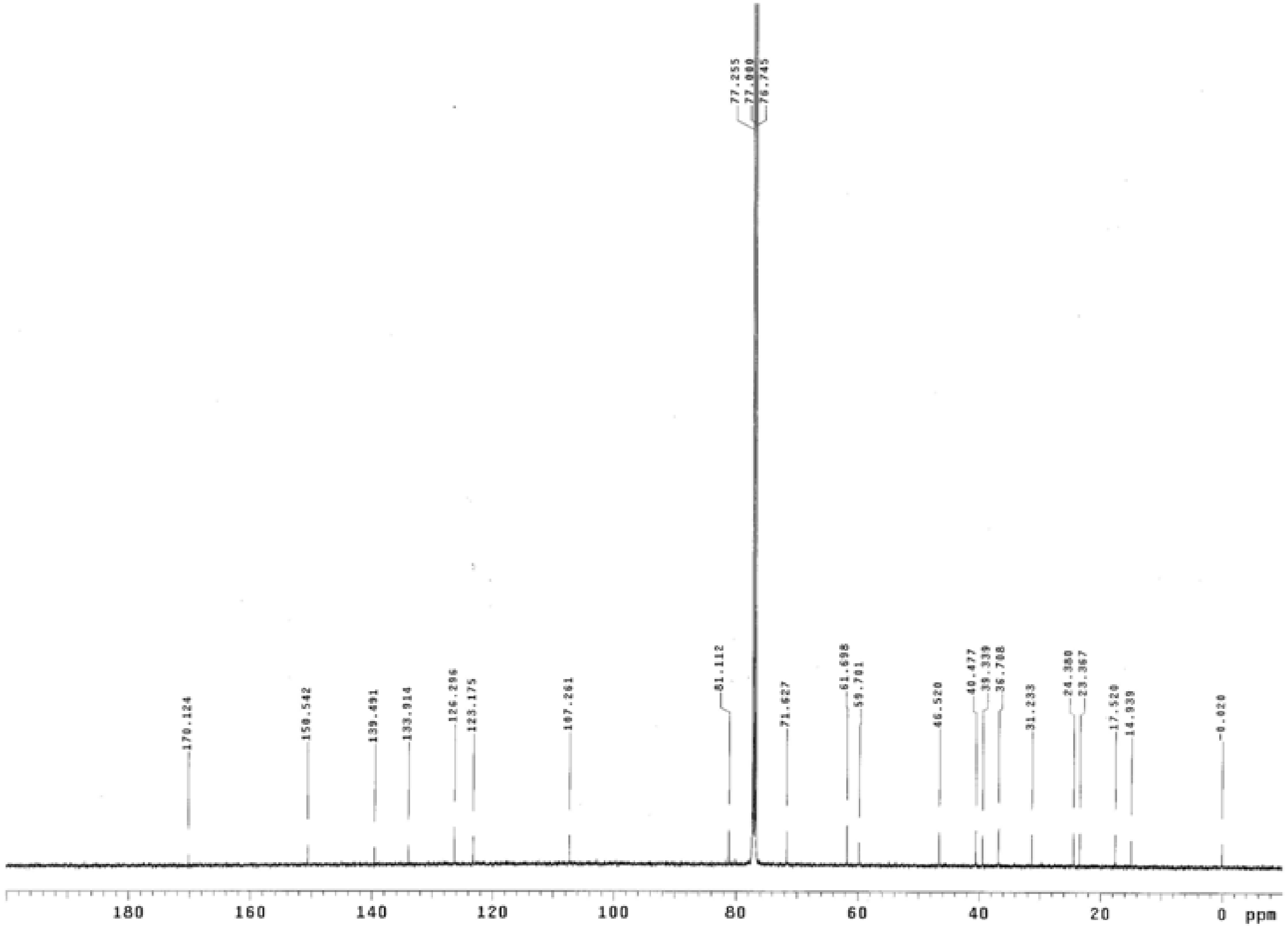


© 2014 by the authors; licensee MDPI, Basel, Switzerland. This article is an open access article distributed under the terms and conditions of the Creative Commons Attribution license (http://creativecommons.org/licenses/by/3.0/).
